# Comparative Study of the Effectiveness of Oral Fluconazole and Intravaginal Clotrimazole in the Treatment of Vaginal Candidiasis

**DOI:** 10.1155/S1064744995000238

**Published:** 1995

**Authors:** Hiroshige Mikamo, Koji Izumi, Kunihiko Ito, Teruhiko Tamaya

**Affiliations:** Department of Obstetrics and Gynecology School of Medicine Gifu University 40, Tsukasa-machi Gifu 500 Japan

## Abstract

*Objective:* A study was carried out to compare 3 treatment regimens for vaginal candidiasis.

*Methods:* A total of 150 women with clinical and mycological evidence of vaginal candidiasis were randomized to receive 50 mg of oral fluconazole daily for 6 days (50 women), a single oral 150 mg dose of fluconazole (50 women), or 100 mg of intravaginal clotrimazole daily for 6 days (50 women). They were assessed at 5–15 days (short-term assessment) and again at 30–60 days (long-term assessment) after the completion of treatment.

*Results:* *Candida* species were completely eradicated from the vagina in 88% or 80% in the 6-day oral fluconzaole group, 76% or 70% in the single oral fluconazole group, and 72% or 60% in the intravaginal clotrimazole group at short-term or long-term assessment, respectively. The rates of clinical effectiveness were 92% or 88% in the 6-day oral fluconzaole group, 80% or 76% in the single oral fluconazole group, and 72% or 58% in the intravaginal clotrimazole group at the short-term or long-term assessment, respectively. Treatment-related side effects were not found in any group.

*Conclusions:* This study suggests that the treatment of vaginal candidiasis with oral fluconazole is effective and that a single oral fluconazole dose might be one choice in the treatment of vaginal candidiasis.


Infectious Diseases in Obstetrics and Gynecology 3:7–11 (1995)
© 1995 Wiley-Liss, Inc.

### Comparative Study of the Effectiveness of Oral Fluconazole and Intravaginal Clotrimazole in the Treatment of Vaginal Candidiasis

**Hiroshige Mikamo, Koji Izumi, Kunihiko Ito, and Teruhiko Tamaya**

*Department of Obstetrics and Gynecology, School of Medicine, Gifu University, Gifu, Japan*

### Abstract

***Objective:*** **A study was carried out to compare 3 treatment regimens for vaginal candidiasis.**

***Methods:*** **A total of 150 women with clinical and mycological evidence of vaginal candidiasis were randomized to receive 50 mg of oral fluconazole daily for 6 days (50 women), a single oral 150 mg dose of fluconazole (50 women), or 100 mg of intravaginal clotrimazole daily for 6 days (50 women). They were assessed at 5–15 days (short-term assessment) and again at 30–60 days (long-term assessment) after the completion of treatment.**

***Results:*** ***Candida*** **species were completely eradicated from the vagina in 88% or 80% in the 6-day oral fluconzaole group, 76% or 70% in the single oral fluconazole group, and 72% or 60% in the intravaginal clotrimazole group at short-term or long-term assessment, respectively. The rates of clinical effectiveness were 92% or 88% in the 6-day oral fluconzaole group, 80% or 76% in the single oral fluconazole group, and 72% or 58% in the intravaginal clotrimazole group at the short-term or long-term assessment, respectively. Treatment-related side effects were not found in any group.**

***Conclusions:*** **This study suggests that the treatment of vaginal candidiasis with oral fluconazole is effective and that a single oral fluconazole dose might be one choice in the treatment of vaginal candidiasis.** © 1995 Wiley-Liss, Inc.

Key words

Vaginitis, vaginal infection, candidal therapy, ketoconazole

Vaginal candidiasis is an infection caused by *Candida albicans* or related fungi. The fungi isolated in the vagina in cases of candidiasis include *C. albicans* in 80–90% and others such as *C. glabrata* and *C. tropicalis*.[1]

The usual transvaginal treatment of candidiasis can be messy and displeasing and can induce local irritation, burning, and frequency of micturition. Consequently, compliance with this form of treatment is poor, as women often stop the treatment as soon as their symptoms disappear, but prematurely before eradication of the fungi. Since the rectum is a carrier of the fungi and relapses are common,[2] oral ketoconazole is one choice of therapy.[3–5]

Fluconazole, a new bis-triazole antifungal agent which is well absorbed orally with a long systemic half-life, has the potential for reducing or eliminating episodes of vaginal candidiasis.[6] In animal models, fluconazole has been shown to be more potent than ketoconazole against *Candida* infections.[7–9]

Clotrimazole, which is effective against dermatophyte and other fungal infections,[10] has been frequently used for local treatment. This trial was designed to compare the efficacy of oral fluconazole with that of intravaginal clotrimazole in the treatment of vaginal candidiasis. The primary criteria of efficacy were the mycological response and clinical response assessed at 5–15 days and 30–60 days after the completion of treatment.

Address correspondence/reprint requests to Dr. Hiroshige Mikamo, Department of Obstetrics and Gynecology, School of Medicine, Gifu University, 40, Tsukasa-machi, Gifu City, Gifu 500, Japan.

Received June 27, 1994

Accepted April 11, 1995

### MATERIALS AND METHODS

### Study Design

An open, comparative study was conducted in the Department of Obstetrics and Gynecology, School of Medicine, Gifu University, Japan. Patients with vaginal candidiasis who agreed to participate in this study were enrolled.

### Patients

A total of 150 women with clinical and mycological evidence of vaginal candidiasis were randomly allocated to receive treatment with 50 mg of oral fluconazole daily for 6 days (50 women), a single oral 150 mg dose of fluconazole (50 women), or 100 mg of intravaginal clotrimazole daily for 6 days (50 women). The clinical diagnosis was initially based on the presence of pseudohyphae (pseudomycelia) seen in the vaginal discharge with the light microscope and subsequently confirmed by culture on Sabouraud chloramphenicol medium (Nissui Pharmaceutical Co., Ltd., Japan). All patients were symptomatic with at least vaginal pruritus or burning. Patients with any clinically relevant associated disease were excluded, as were patients who were pregnant or lactating or known to have impaired hepatic or renal function. Women with venereal disease, chlamydial infection, trichomoniasis, or vaginitis other than candidiasis were also excluded.

### Efficacy Assessment

The mycological examination of the vaginal discharge was performed with 2 vaginal swabs: one was inoculated in a transport medium for *Trichomonas* or bacteria, and the second was transported for fungal culture on a Sabouraud chloramphenicol agar (Nissui Pharmaceutical Co., Ltd.) and identification of the species, after a 48-h incubation, with an API 20C Auxanogramm (Bio Merieux, France). *C. albicans* was distinguished from other species such as *C. tropicalis, C. glabrata, C. parapsilosis,* and *C. krusei*. For the bacteriological study, a vaginal examination was performed and a sample of vaginal discharge was collected before therapy was started. Simultaneously, a sample was taken with a polyester fiber swab (Falcon Applicator, Becton Dickinson, Cockeysville, MD) and suspended in 5 ml of an anaerobic buffer containing a reducing agent in a $CO_2$-filled tube. The composition of the anaerobic buffer was $KH_2PO_4$: 4.0 g; $Na_2HPO_4$: 6.0 g; L-cysteine · $HCl \cdot H_2O$: 1.0 g; Tween 80 (Sigma Chemical Company, St. Louis, MO): 1.0 g; agar: 1.0 g; and distilled water: 1,000 ml/pH 7.2. All the components were mixed and then heated at 80°C for 30 min to produce a solution. A 9 ml quantity of the buffer was transferred to a test tube. Immediately after the air in the tube was replaced with $CO_2$, the tube was sealed with a butyl rubber stopper. All tubes and solutions were sterilized in an autoclave at 115°C for 20 min. After the sample was suspended in the buffer, the tube was resealed under a continuous stream of carbon dioxide gas of commercial grade to drive the air out. Exposure of the sample to atmospheric oxygen was restricted to no longer than 5 min. The samples were suspended in the solution and immediately incubated. For *T. vaginalis,* the simple *Trichomonas* "Fuji" medium (Fuji Pharmaceutical Co., Ltd., Japan) was used. The DNA probe method was used for the detection of chlamydial infection only in cases in which the macroscopic findings suggested such an infection. These examinations were undertaken at the baseline, short-, and long-term assessments. Fungal eradication was defined as both a negative culture and negative microscopy for *Candida* after treatment. If other pathogens such as *T. vaginalis, Gardnerella vaginalis,* or *Chlamydia* species were detected before treatment, the patient was excluded from the efficacy analysis to assure an accurate evaluation of the therapeutic efficacy against candidiasis.

The symptoms and signs of vaginal infection were recorded before treatment. The significant parameters for the clinical assessment were subjective symptoms (genital itching and genital discharge) and objective vaginal findings (state of skin and genital discharge). The clinical assessment of effectiveness was made by a doctor and the patient at 5–15 days (short-term assessment) and again at 30–60 days (long-term assessment) after the last treatment. The clinical outcome was based upon a clinical cure (complete disappearance of presenting signs and symptoms) and symptomatic improvement. Each symptom or sign was assessed by the same investigator. The statistical analysis was performed by U-test.

### Safety Assessment

The side effects were recorded during treatment. Routine laboratory tests were carried out at the baseline and short-term assessments.

**TABLE 1. Comparison of mycological effectiveness of oral fluconazole and intravaginal clotrimazole in the treatment of vaginal candidiasis**

| Regimen | No. | Clinical effectiveness (negative fungi) (%) 5th–15th day assessment | 30th–60th day assessment |
|---|---|---|---|
| 50 mg of oral fluconazole daily for 6 days | 50 | 88 | 80 |
| Single oral 150 mg dose of fluconazole | 50 | 76 | 70 |
| 100 mg of intravaginal clotrimazole daily for 6 days | 50 | 72 | 60 |

### RESULTS

### Patients

The mean age of all patients was 32.5 years (range 18–54 years) and the mean body weight was 56.5 kg (range 41.0–77.5 kg). In the 6-day oral fluconazole group, the mean age of the patients was 30.4 years (range 20–48 years) and the mean body weight was 56.5 kg (range 41.0–71.0 kg). In the oral 150 mg fluconazole group, the mean age of the patients was 35.0 years (range 18–50 years) and the mean body weight was 56.4 kg (range 43.2–77.5 kg). In the group receiving 100 mg of intravaginal clotrimazole for 6 days, the mean age of the patients was 32.1 years (range 21–54 years) and the mean body weight was 56.6 kg (range 41.5–69.5 kg).

### Efficacy

A comparison of the mycological effectiveness of oral fluconazole and intravaginal clotrimazole in the treatment of vaginal candidiasis is shown in Table 1. *Candida* species were completely eradicated from the vagina in 88% or 80% in the 6-day oral fluconazole group, 76% or 70% in the single oral fluconazole group, and 72% or 60% in the intravaginal clotrimazole group at the short-term or long-term assessment, respectively.

A comparison of the clinical effectiveness of oral fluconazole and intravaginal clotrimazole in the treatment of vaginal candidiasis is shown in Table 2. The rate of clinical effectiveness was obtained in 92% or 88% in the 6-day oral fluconazole group, 80% or 76% in the single oral fluconazole group, and 72% or 58% in the intravaginal clotrimazole group at the short-term or long-term assessment, respectively. A significant difference was not noted in the different therapies.

### Safety

Neither adverse side effects to the drug nor abnormal laboratory values were noted during the treatment or observation periods.

### DISCUSSION

The symptoms of vaginal candidiasis vary from irritation and discharge in a mild infection to perineal discomfort and dyspareunia in a severe infection. The long-term cure of vaginal candidiasis is clinically significant in view of its high recurrence rate and frequent relapses after conventional topical therapy. Topical treatment, although effective, is associated with poor compliance and premature discontinuation of the treatment.[11] The relapses associated with vaginal fungal infections are also related to the difficulty of completely eradicating fungi from the vagina in certain physiological conditions. Moreover, a single negative culture of the vaginal lumen does not guarantee that the organisms have been totally eliminated. Since *Candida* species invade the tissues, systemic treatment with fluconazole could eliminate *Candida* from the vaginal tissues.

The rectum, an important reservoir of *Candida* species, may also be responsible for relapses. In a previous study on vaginal candidiasis, we detected *C. albicans* in 35% of women with first-episode candidiasis and 46% of women with recurrent candidiasis. Since intravaginally administered antifungal agents are minimally absorbed in the body and are unlikely to influence the rectal flora, the long-term effectiveness noted with oral fluconazole might reflect a concomitant reduction of rectal fungal flora.

In comparing the effectiveness of these 3 regimens of treatment, we found each regimen to be

**TABLE 2. Comparison of clinical effectiveness of oral fluconazole and intravaginal clotrimazole in the treatment of vaginal candidiasis**

| | | Clinical effectiveness | | | |
|---|---|---|---|---|---|
| Regimen | No. | 5th–15th day assessment (%) | Statistical analysis | 30th–60th day assessment (%) | Statistical analysis |
| 50 mg of oral fluconazole daily for 6 days | 50 | 92 | N.S.[a] | 88 | N.S. |
| Single oral 150 mg dose of fluconazole | 50 | 80 | N.S. | 76 | N.S. |
| 100 mg of intravaginal clotrimazole daily for 6 days | 50 | 72 | N.S. | 58 | N.S. |

[a]N.S. = not significant.

very effective in the treatment of vaginal candidiasis. A significant difference was not noted in the different therapies, which may be attributable to the small number of patients or poor statistical power of this study. However, oral fluconazole might be more effective in short-term and long-term assessments, as suggested in previous studies.[12–16] At the same time, fluconazole, even as a single oral dose, is effective and reliable in the treatment of vaginal candidiasis. In addition, the symptoms of candidiasis are rapidly relieved by oral treatment.[17,18]

In the United States, a comparison of drug treatment costs for vaginal candidiasis revealed that the single dose treatment with fluconazole (150 mg) is less expensive than intravaginal treatment with clotrimazole, miconazole, or terconazole.[19] On the contrary, in Japan, the national health insurance (NHI) price of 150 mg of fluconazole is 2,882 yen, while a 6-day course of intravaginal clotrimazole, 100 mg daily, costs 477.6 yen. Nevertheless, cost reductions resulting from decreases in physician office visits and associated laboratory tests may result if recurrences and relapses can be reduced with fluconazole treatment.

The results of our study suggest that the treatment of vaginal candidiasis with oral fluconazole is effective and that a single oral fluconazole dose might be one choice in the treatment of vaginal candidiasis.

### REFERENCES

1. Odds FC: Genital candidiasis. Clin Exp Dermatol 7:345–354, 1982.
2. Miles MR, Olsen L, Rogers A: Recurrent vaginal candidiasis. Importance of an intestinal reservoir. JAMA 238:1836–1837, 1977.
3. Hawkins Van Tyle J: Ketoconazole. Pharmacotherapy 4:343–373, 1984.
4. Bisschop MPJM, Merkus JMWM, Scheygrond H, Van Cutsem J, Van de Kuy A: Treatment of vaginal candidiasis with ketoconazole, a new orally active antimycotic. Eur J Obstet Gynaecol Reprod Biol 9:253–258, 1979.
5. Van der Pas H, Peeters F, Janssens D, Schauwaert E, Van Cutsem J: Treatment of vaginal candidiasis with oral ketoconazole. Eur J Obstet Gynaecol Reprod Biol 14: 399–404, 1983.
6. Humphrey MJ, Jevons S, Tarbit MH: Pharmacokinetic evaluation of UK-49,858, a metabolically stable triazole antifungal drug, in animals and humans. Antimicrob Agent Chemother 28:648–653, 1985.
7. Richardson K, Brammer KW, Marriott MS, Troke PF: Activity of UK-49,858 (fluconazole), a bistriazole derivative, against experimental infections with *Candida albicans* and *Trichophyton mentagrophytes*. Antimicrob Agents Chemother 27:832–835, 1985.
8. Shaw JJB, Tarbit MH, Troke PF: Cytochrome P-450 mediated sterol synthesis and metabolism: Differences in sensitivity to fluconazole and other azoles. In Fromtling RA (ed): Recent Trends in the Discovery, Development and Evaluation of Antifungal Agents. Barcelona: JR Prous Publishers, pp 125–139, 1987.
9. Troke PF, Andrews RJ, Brammer KW, Marriott MS, Richardson K: Efficacy of UK-49,858 (fluconazole) against *Candida albicans* experimental infections in mice. Antimicrob Agents Chemother 28:815–818, 1985.
10. Bisschop MPJM, Merkus JMWM, Scheygrond H, van Cutsem J: Patients' preference of oral treatment in vaginal candidiasis (a double-blind study). J Drug Ther Res 10: 587–589, 1985.
11. O'Connor MI, Sobel JD: Epidemiology of recurrent vulvovaginal candidiasis: Identification and strain differentiation of *Candida albicans*. J Infect Dis 154:358–363, 1986.
12. Osinusi BO, Rotowa NA: Fluconazole as single-dose treatment of vulvo-vaginal candidosis. Curr Ther Res 43(6): 1014–1018, 1988.
13. Ree T, Phillips R: Multicentre comparison of one-day oral therapy with fluconazole or itraconazole in vaginal candidiasis. Int J Gynaecol Obstet Suppl 37:33–38, 1992.
14. Kutzer E, Oittner R, Leodolter S, et al.: A comparison of fluconazole and ketoconazole in the oral treatment of vaginal candidiasis: Report of a double-blind multicentre trial. Eur J Obstet Gynaecol Reprod Biol 29:305–313, 1988.

15. Stein GE, Christensen S, Mummaw N: Comparative study of fluconazole and clotrimazole in the treatment of vulvovaginal candidiasis. DICP 25:582–585, 1991.
16. Oral fluconazole for vaginal candidiasis. Med Lett Drugs Ther 36(931):81–82, 1994.
17. Brammer KW, Lees LJ: Single dose oral fluconazole in the treatment of vulvo-vaginal candidiasis: An interim analysis of comparative study versus three-day intra-vaginal clotrimazole tablets. In Fromtling RA (ed): Recent Trends in the Discovery, Development and Evaluation of Antifungal Agents. Barcelona: JR Prous Publishers, pp 151–156, 1987.
18. Multicentre Study Group (responsible author: Brammer KW): Treatment of vaginal candidiasis with a single oral dose of fluconazole. Eur J Clin Microbiol Infect Dis 6:364–367, 1988.
19. Phillips RJM, Watson SA,McKay FF: An open multicentre study of the efficacy and safety of a single dose of fluconazole 150 mg in the treatment of vaginal candidiasis in general practice. Br J Clin Pract 44:219–222, 1990.
